# Reduction of lipoxidative load by secretory phospholipase A2 inhibition protects against neurovascular injury following experimental stroke in rat

**DOI:** 10.1186/1742-2094-6-21

**Published:** 2009-08-13

**Authors:** Md Nasrul Hoda, Inderjit Singh, Avtar K Singh, Mushfiquddin Khan

**Affiliations:** 1 Department of Pediatrics, Medical University of South Carolina, Charleston, SC 29425, USA; 2 Department of Pathology and Laboratory Medicine, Ralph J Johnson VA Medical Center, Charleston, SC 29425, USA

## Abstract

**Background:**

In animal models, ischemia reperfusion (IR) injury triggers membrane lipid degradation and accumulation of lipoxidative exacerbations in neurovascular unit, leading to blood brain barrier (BBB) damage and neurologic deficits. In this study, we investigated whether impeding membrane lipid breakdown by inhibiting secretory phospholipase A2 (sPLA2) activity reduces BBB leakage, leading to neuroprotection and functional recovery.

**Methods:**

Focal cerebral IR injury was induced by middle cerebral artery occlusion (MCAO) in adult male rats. A sPLA2 inhibitor, 7,7-dimethyleicosadienoic acid (DEDA), was administered following IR injury. DEDA-treated animals were compared with vehicle-treated in terms of BBB leakage, edema, infarct volume, and neurological deficit. Membrane lipid degradation and the expression/activity of sPLA2 were also assessed. The role of one of the sPLA2 products, arachidonic acid (AA), on the morphology of the differentiated neuronal cell PC12 was examined by light microscopy.

**Results:**

Treatment with DEDA after IR injury not only reduced BBB leakage but also decreased infarct volume and improved neurologic function. The treatment attenuated both the activity of sPLA2 and the levels of sPLA2-derived oxidized products. The metabolites of lipid oxidation/peroxidation, including the protein carbonyl, were reduced as well. The treatment also restored the levels of glutathione, indicating attenuation of oxidative stress. I*n vitro *treatment of PC12 cells with DEDA did not restore the AA-mediated inhibition of neurite formation and the levels of glutathione, indicating that effect of DEDA is up stream to AA release.

**Conclusion:**

sPLA2-derived oxidative products contribute to significant neurovascular damage, and treatment with sPLA2 inhibitor DEDA ameliorates secondary injury by reducing exacerbations from lipoxidative stress.

## Background

Stroke involves a myriad of biochemical events [1]. Following cerebral ischemia-reperfusion (IR) injury, membrane lipid degradation, reactive oxygen species (ROS) generation, glutamate excitotxicity and calcium overload are the major initial events that induce inflammation and cellular death [2]. Excess release of calcium and glutamate results in phospholipid hydrolysis and the release of arachidonic acid (AA) via phospholipase activation [3]. Phospholipids are major lipid components crucial for membrane integrity and synaptic function. Degraded lipids compromise not only the structural integrity of the cellular membrane but also produce reactive oxygen species (ROS), overwhelming the antioxidant system in the neurovascular unit [4].

ROS, generated through several cellular pathways following IR, have been implicated in neuronal dysfunction [5]. Increased vulnerability of the brain to lipoxidative mediators following IR injury contributes to secondary injury and impairment of brain functions [6]. Glutathione (GSH), an endogenous antioxidant that reduces ROS levels, plays a central role within the finely tuned network of antioxidant systems that respond to insult. GSH responds to oxidative stress through its peroxide scavenging functions via glutathione S-transferase (GST) and glutathione peroxidase (GPx) [2]. Accordingly, the GSH precursor N-acetylcysteine (NAC) provided neurovascular protection following IR injury [7], supporting the potential of reducing ROS strategy for stroke [6].

Understanding the effects of lipoxidative load on the mechanisms of pro-inflammatory enzymes, like phospholipases A2 (PLA2s), and their modulation for therapeutic purposes has gained significant recent attention [8,9]. Among the different types of PLA2s, secretory PLA2s (sPLA2s) play an important role in neuroinflammation due to their non-specific nature to phospholipid substrate [10,11]. Cytosolic PLA2 (cPLA2) will specifically release AA; while sPLA2 does not have this specificity of fatty acid too [12]. Due to their accessibility in the circulation and in the tissues, sPLA2s activity leads to generation of several potent mediators of inflammation. The products of sPLA2, free fatty acids (FFA) including AA and lysophosphatidylcholine (LPC), are the most crucial bioactive lipid metabolites. AA, which induces oxidative stress during its metabolism, leads to either cell proliferation or apoptosis, depending on the cell type in which it is metabolized [13-16]. AA also regulates downstream signaling pathways of p38 mitogen-activated protein kinase (MAPK) [17-19], which is involved in the disruption of the blood brain barrier (BBB) [20]. Furthermore, released free AA either acts as a second messenger or it is further metabolized by relevant enzymes to generate eicosanoid signaling molecules like prostaglandins, leukotrienes and thromboxanes [21]. These, in turn, set the stage for oxidative and peroxidative damage to cellular membranes [22,23].

Recent reports have shown that sPLA2s are active before secretion from the cell [6] and can be re-internalized into cells viacaveolae in which localized Ca^2+ ^concentrations may be sufficiently high to permit lipid turnover. Induction of sPLA2 triggers activation of cytosolic PLA2 (cPLA2) via its receptor mediated internalization into the cytosol and regulates the invasiveness of matrix metalloproteinases (MMPs) [24]. MMPs have been reported to aggravate IR injury through hemorrhagic transformation and BBB disruption in the acute phase of stroke [25].

Membrane lipid degradation by sPLA2 and the consequent lipoxidative metabolism have been documented as crucial toxic mechanisms involved not only in IR injury but also in other neurodegenerative diseases [8,26-28], making these processes potential therapeutic targets for neurovascular protection [29,30]. Increased expression of sPLA2 has been reported in cerebral ischemia [12,31], and sPLA2 inhibition provides protection against ischemic injury [32,33]. Among different subtypes of sPLA2, group IIA has been extensively studied [11,34], and inhibition of this sPLA2 has been reported to reduce lesion volume following IR injury [9]. However, the role of sPLA2 and its metabolic products in BBB disruption is not understood.

A selective inhibitor of sPLA2 IIA [35], 7,7-dimethyleicosadienoic acid (DEDA) is a non-toxic AA analog with IC_50 _values in the range of 6–20 μM. DEDA has been documented to reduce sPLA2 activity significantly and decrease carotid artery ischemia-evoked release of glutamate and aspartate in the cerebral cortex when administered after injury in rats [36]. Treatment with DEDA has shown no effect on physiologic parameters, including blood pressure, pH and blood gases [37]. However, it significantly reduced levels of lipid metabolites including LPC and 6-keto-PGF1 alpha [36]. Recently, we documented the therapeutic potential of DEDA against the psychosine-mediated cellular toxicity implicated in the pathology of Krabbe disease, a demyelinating neurodegenerative disease. Inhibition of sPLA2 by DEDA reduced the levels of AA and LPC, resulting in the survival of oligodendrocytes after treatment with psychosine [38]. These studies indicate the potential of DEDA to reduce the sPLA2-derived lipoxidative exacerbations so deleterious in the neurovascular unit following IR injury.

## Methods

### Reagents and cell culture

Dulbecco's Modified Eagle's Medium (DMEM) with glucose, L-glutamine and sodium pyruvate was purchased from Mediatech, Inc. (Herndon, VA). Fetal Bovine Serum (FBS) and Hank's balanced salt solution (HBSS) were obtained from Life Technologies (Carlsbad, CA). 2,3,5-triphenyltetrazolium chloride (TTC), 3,4,5-dimethylthiazol-2-yl-2, 5-diphenyltetrazolium bromide (MTT) and 1-chloro 2,4-dinitrobenzene (CDNB) were obtained from Sigma-Aldrich Chemical Corporation (St. Louis, MO). Antibodies against sPLA2-IIA and CD34+ were purchased from Santa Cruz Biotechnology, Inc. (Santa Cruz, CA). DEDA was purchased from Biomol International L.P. (Plymouth Meeting, PA, USA). 1-palmitoyl-2-[1-^14^C] arachidonyl-sn-glycero-3-phosphocholine and – [1-^14^C] arachidonic acid were obtained from American Radiolabeled Inc., St. Louis, MO.

### Experimental animals

All animal procedures were approved by the Medical University of South Carolina (MUSC)'s Animal Review Committee, and animals received humane care in compliance with MUSC's experimental guidelines and the National Research Council's criteria for humane care (Guide for the Care and Use of Laboratory Animals).

### Experimental design and administration of drugs

The animals were divided into four groups: (i) control (sham-operated) group (ii) ischemia (60 minutes) and reperfusion (24 hr) group (Vehicle or Veh), (iii) DEDA-treated at reperfusion and repeated at 3 h of reperfusion group (DEDA), and (iv) DEDA-treated at reperfusion group. In the treatment group, DEDA (1 mg/kg body weight) dissolved in sterile DMSO (25 μl) was slowly infused via tail vein. Rats in the vehicle and sham groups were administered the same volume of DMSO.

### Focal cerebral ischemia

Male Sprague-Dawley rats weighing 250–300 g (Harlan Laboratories, Wilmington, MA) were fasted overnight but allowed access to water *ad libitum*. They were anesthetized with an intramuscular injection of xylazine (10 mg/kg body weight) and an intraperitoneal injection of ketamine hydrochloride (100 mg/kg). A rectal temperature probe was introduced, and a heating pad was used to maintain the body temperature at 37°C. Right middle cerebral artery (MCA) was occluded as described by Longa et al [39] with modifications [40,41]. Briefly, focal cerebral ischemia was induced by introducing a 4 cm long silicone coated (coating length 5 mm and 0.35–0.37 mm coating diameter) specialized occluding suture for MCAO (Doccol Corporation, Redlands, CA; Cat# 4035 or 4037, as per the weight of the animal) into the internal carotid artery (ICA) via external carotid artery (ECA) stump until the suture was wedged, and the tip occluded the proximal stem of MCA, approximately internalizing 20 mm of total length. 60 minutes post occlusion, the filament was withdrawn to allow reperfusion and the ECA stump was ligated and coagulated permanently. The animals were euthanized by decapitation under deep anesthesia with a pentobarbital overdose (150 mg/kg) at the specified time period of reperfusion to harvest brain for biochemical estimations or for immunohistochemical examination. The brains were snap-frozen and stored at -70°C.

### Measurement of physiologic parameters

The physiologic parameters were measured before 30 min of MCAO and at 3.5 h after reperfusion (ie. 30 min after DEDA treatment) and are presented in Table 1. Mean blood pressure (MBP) and heart rate (HR) and blood pH were measured without anesthesia. The rectal temperature was monitored and maintained at about 37 to 37.8°C. Body temperature was monitored by a probe maintained at about 37 ± 0.5°C by a homeothermic blanket control unit (Harvard Apparatus, Holliston, MA). Cranial temperature was measured by HSE Plugsys TAM-D (Harvard Apparatus). Blood pH was measured by pH/blood gas analyzer iSTAT (Heska, Fort Collins, CO). MBP and HR were measured using a XBP1000 NIBP system (Kent Scientific, Torrington, CT). It is non-invasive computerized tail-cuff system and uses automated inflation/deflation pump.

**Table 1:** Physiologic Parameters

	VEH		DEDA	
	Basal	3.5 h Rep	Basal	3.5 h Rep

Rectal Temp (°C)	37.0 ± 0.1	36.4 ± 0.2	37.8 ± 0.1	37.5 ± 0.1

Cranial Temp (°C)	37.2 ± 0.7	37.5 ± 0.8	37.8 ± 0.7	37.9 ± 0.9

MBP (mm Hg)	120.97 ± 3.93	121.1 ± 1.94	121.5 ± 4.0	129.1 ± 6.39

HR	355.5 ± 17.1	364.4 ± 21.39	362.3 ± 17.23	378.2 ± 8.67

pH	7.2 ± 0.2	7.4 ± 0.2	7.4 ± 0.3	7.4 ± 0.2

Measurements were performed before MCAO (base) and at 3.5 h after reperfusion without anesthesia as described in Methods. pH was measured in blood also at 3.5 h after reperfusion. Data are presented as mean ± SD for n = 3 in each group. Measurements were also performed for sham group. No significant differences were observed among the groups. Basal, 30 min before MCAO. HR, heart rate; MCAO, middle cerebral artery occlusion; MBP, mean blood pressure; Rep, reperfusion; Temp, temperature

### Evaluation of neurologic deficit

Neurologic deficits in the animals were assessed by an observer blinded to the identity of the groups after 60 min of ischemia and 24 h of reperfusion. The scoring was based on the method of Huang et al. [42] and previously adopted by us [43].

### Measurement of ischemic infarct

Infarct volume was evaluated as described previously [44]. Briefly, after 24 h of reperfusion, the brain was quickly removed, washed in ice-cold phosphate buffered saline (PBS) and 2 mm coronal sections were obtained to incubate with 2% TTC dissolved in saline for 20 min at 37°C. After washing with chilled PBS for 5 min, images were made and acquired in Photoshop 7.0 (Adobe Systems). The infarct area was quantified using Scion image, an image-analysis software program (Scion Corporation).

### Quantitation of FFA, AA and LPC in the ipsilateral hemisphere of brain

Lipids were extracted from the ipsilateral hemisphere of brain tissue by the Folch method as described earlier [45,46]. FFA was determined and quantified using high performance thin layer chromatography (HPTLC) plates [46]. AA present in FFA was measured using capillary gas chromatography (GC) as described by Rao and colleagues [47]. Quantification of LPC was performed by one-dimensional HPTLC (LHPK from Whatman, Inc.; Florham Park, NJ) using the method described by Weerheim et al. [48], with modification. Briefly, plates were developed in methyl acetate-1-propanol-chloroform-methyl alcohol-0.25% KCl-acetic acid (100:100:100:40:36.5:2; v/v/v/v/v/v) and visualized by heating at 200°C for 6 min after spraying with 10% CuSO_4 _in 8% phosphoric acid. Different concentrations (0.2 to 5.0 mg) of LPC (1-palmitoyl LPC) were resolved on the same plate as standard for quantification. LPC was quantified by densitometric scanning using the Imaging Densitometer (model GS-670; Bio-Rad).

### Measurement of PLA2 Activity

PLA2 activity was measured as reported earlier [49]. Brain tissue was homogenized in 10 mM HEPES buffer (pH 7.2) containing 0.5 mM each of EGTA, EDTA and a 1× protease inhibitor cocktail. The homogenate was centrifuged at 18, 000 × g, and the supernatants were collected. PLA2 activity was determined as the release of [1-^14^C] arachidonic acid from 1-palmitoyl-2-[1-^14^C] arachidonyl-sn-glycero-3-phosphocholine.

### cDNA synthesis and real time PCR for mRNA expression of sPLA2

cDNA synthesis and real time PCR analysis were carried out as described earlier [50]. Total RNA from brain tissue was isolated using Trizol reagent (Gibco BRL, Carlsbad, CA) per manufacturer's instructions. Single-stranded cDNA was synthesized from RNA samples of rat brains by using the superscript preamplification system (Life Technologies, Carlsbad, CA). Quantitative real-time PCR was performed with the Bio-Rad (Hercules, CA) iCycler iQ Real-Time PCR Detection System per conditions described previously [50]. Briefly, primer sets were designed and obtained from Integrated DNA Technologies (IDT, Coralville, IA). The primer sequences were: GAPDH, forward primer, 5'-CCTACCCCCAATGTATCGTTGTG-3', reverse primer, 5'-GGAGGAATGGAGTTGCTGTTGAA-3'; sPLA2-IIA, forward primer, 5'-GTGACTCATGACT GTTGTTAC-3', reverse primer, 5'-CAAAACATTCAGCGGCAGC-3'. Thermal cycling conditions were as follows: activation of iTaq DNA polymerase at 95°C for 10 minutes, followed by 40 cycles of amplification at 95°C for 30 seconds and 55–57.5°C for 1 minute. The normalized expression of the target gene with respect to GAPDH was computed for all samples by using Microsoft Excel data spreadsheets.

### Immunohistochemistry

Protein expression of sPLA2 was detected by immunohistochemical analysis. In brief, the brain tissue sections were de-parafinized and re-hydrated in sequential gradations of alcohol. After antigen unmasking in unmasking solution (Vector Labs, CA), sections were cooled and washed three times for two minutes each in PBS. Sections were immersed for 10 min in 3% hydrogen peroxide to eliminate endogenous peroxidase activity and blocked in 1% bovine serum albumin for 1 hr. Sections were incubated overnight with a primary antibody (Santa Cruz Biotechnology, CA; 1:100 dilutions in blocking buffer). After washing in PBS containing 0.1% Tween-20, sections were incubated with a fluorophore tagged secondary antibody (1:100 dilutions in blocking buffer) (Vector Labs, CA). Fluorescence was visualized under the microscope. All the sections were analyzed using a Zeiss Olympus Microscope, and images were captured using a Kontron Digital Camera. Different fields were recorded from different sections, and representative images were presented in figures. Images were captured and processed in Adobe Photoshop 7.0 and were adjusted for brightness, contrast and unmasking tools to enhance image clarity.

### Measurement of protein carbonyl (PC) in brain tissue

Content of protein carbonyl was determined using the method reported by Levine et al [51] with certain modifications. Tissue homogenates, prepared in chilled 20 mM PBS (pH 7.4) containing 1× protease inhibitor cocktail, were centrifuged at 2000 × g for 3–5 minutes to remove debris. The supernatant was taken and incubated with streptomycin sulfate (final concentration 1%) for 15 min at room temperature, followed by centrifugation at 2800 × g for 5 min. 100 μl of supernatant was taken in duplicates, and the protein was precipitated by adding 500 μL of 20% chilled TCA. The mixture was centrifuged at 3000 × g, and the supernatant was discarded. The pellet was dissolved in 800 μL of 20 mM DNPH solution prepared in 2 M HCl, and incubated at room temperature in the dark for 1 hr with overtaxing every 15 min. Negative controls were run in duplicates for each sample by adding 800 μL of 2 M HCL in place of DNPH solution. After one hr, 700 μl of 20% chilled TCA was added and kept on ice-bath for 5 – 10 minutes. The suspension was centrifuged at 10,000 × g for 10 min, and the supernatant was discarded. The pellets obtained were washed with 3 × 1 ml by ethanol – ethyl acetate mixture (1:1). Finally, they were dissolved at 50°C in 1 ml of 6 M guanidine hydrochloride solution prepared in 0.1 M potassium phosphate buffer. The supernatant was obtained by centrifugation at 10,000 × g. The yellow colored complexes obtained were read vs. negative controls at 370 nm to determine the amount of PC. The negative controls were read against 6 M-guanidine solution at 280 nm to determine protein concentrations.

### Measurement of reduced glutathione (GSH) in brain tissue

Concentration of glutathione (GSH) was measured using a colorimetric assay kit for glutathione from Oxis Research (Portland, OR) as reported earlier [41]. Briefly, tissues were minced and homogenized (20 ml/g tissue in 5% metaphosphoric acid). Homogenates were centrifuged at 3,000 × *g *for 10 min. Supernatants were used to assay GSH at 400 nm as described previously [41].

### Evaluation of blood brain barrier (BBB) disruption by Evan's blue (EB) extravasation and measurement of edema

BBB leakage was assessed by the method of Weismann and Stewart [52] with slight modification. The rats received 100 μl of a 5% solution of EB in saline administered intravenously after DEDA treatment. At the completion of reperfusion time, cardiac perfusion was performed under deep anesthesia with 200 ml of saline to clear the cerebral circulation of EB. The brain was removed, sliced and photographed. The two hemispheres were isolated and mechanically homogenized in 750 μl of N, N-dimethylformamide (DMF). The suspension obtained was kept at room temperature in the dark for 72 hr. It was centrifuged at 10,000 × g for 25 minutes, and the supernatant was spectrofluorimetrically analyzed (λ_ex _620 nm, λ_em _680 nm).

At 24 h following MCAO, animals were euthanized to determine brain water content (edema). The cortices, excluding the cerebellum, were quickly removed and the contralateral and the ipsilateral hemispheres separately weighed. Each hemisphere was dried at 60°C for 72 hours and the dry weight was determined. Water content was calculated in ipsilateral hemisphere as: water content (%) = (wet weight – dry weight)/wet weight × 100.

### Maintenance of cell lines and study of morphological changes

PC12 pheochromocytoma cells (a neuronal cell line) were purchased from ATCC (Manassas, VA, USA) and maintained in DMEM (4.5 g glucose/L) supplemented with 10% horse serum, 5% fetal bovine serum and 1% antibiotics. To investigate the effect of AA mediated morphological changes on NGF-induced neuronal differentiation, PC12 cells were initially cultured in the medium mentioned above under 5% CO_2 _in poly-D-lysine-coated plates (Costar, Cambridge, MA). A medium containing DMEM (4.5 g glucose/L), 0.1% bovine serum albumin and 1% antibiotic was used in all differentiation studies. Cells (x10^5^/ml) were plated on 6-well polystyrene tissue culture plates (Costar, Cambridge, MA) and grown for 4 days in serum containing medium. 20 ng/ml NGF was added, which is the dose yielding 50% maximal differentiation of PC12 cells. Neuronal differentiation was observed after 72 hr by morphological parameters, specifically the appearance of axodendritic processes > 40 μm long (about 2–3 cell diameters), using phase contrast microscopy. Under qualified differentiation conditions, cells were labeled with 0.1 μCi of AA. The plates were pre-treated with DEDA for 2 hr, followed by treatment with cytokines and/or H_2_O_2_. Morphological and biochemical alterations were assessed 24 hr later.

### Statistical analysis

Statistical analysis was performed using software Graphpad Prism 3.0, unless otherwise stated. Values are expressed as mean ± SD of n determinations or as mentioned. Comparisons among means of groups were made with a two-tailed Student's *t-test *for unpaired variables. Multiple comparisons were performed using one-way ANOVA followed by the Bonferroni test. P-values < 0.05 were considered significantly different.

## Results

### DEDA improves neurological function and reduces infarct volume

**Figure 1:**
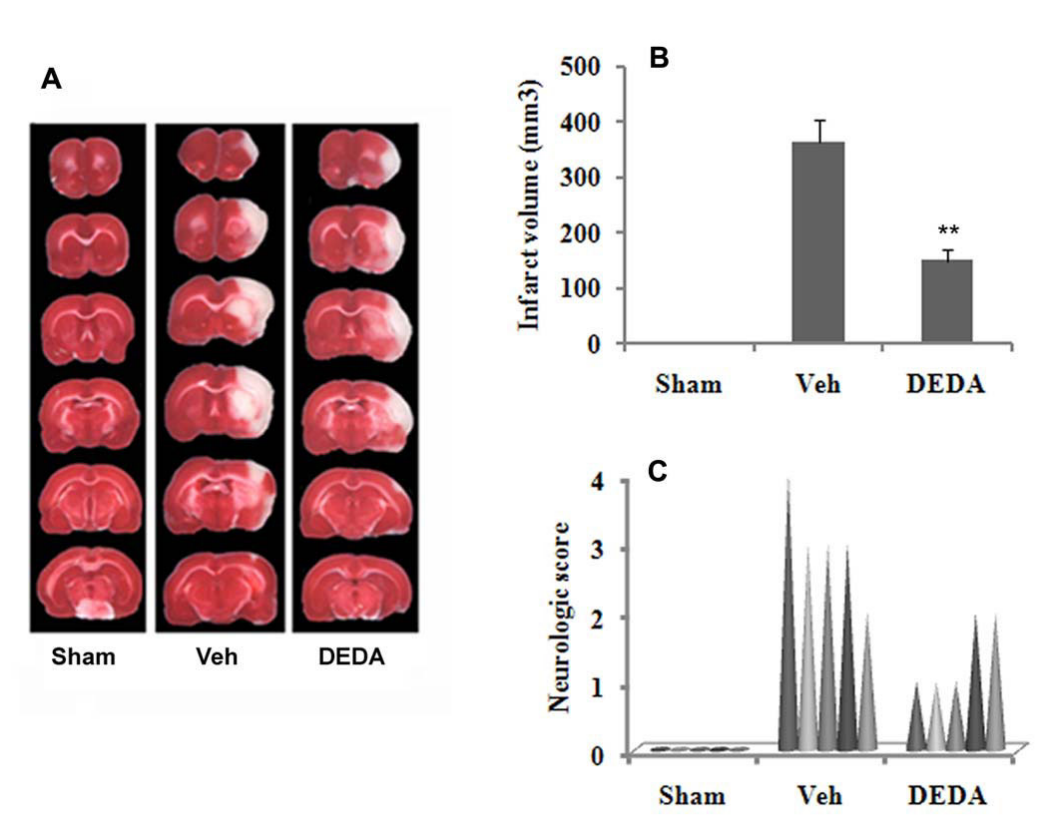
**Post-injury treatment with DEDA protects the brain against IR injury and improves neurologic score**. (A) Photographs showing the effect of DEDA (1 mg/kg) administered at reperfusion and repeated at 3 h following reperfusion after 60 min MCAO on TTC-stained sections, (B) Effect of DEDA on infarct volume (measured in six serial coronal sections arranged from cranial to caudal regions) and (C) Effect of DEDA on neurologic score. Data for infarct volume (n = 5) are presented as means ± SD. Data for neurological deficit (n = 5) are presented as individual data points. ** p < 0.01 vs. vehicle (Veh).

The experimental animals were assessed for neurologic deficit and infarctions at 24 hr of reperfusion following 60 min MCAO. Fig 1A–B shows that IR injury was reduced significantly (p ≤ 0.01) in the DEDA treated at reperfusion and repeated at 3 h of reperfusion group (infarct volume 150 ± 20 mm^3^) compared to the Veh (infarct volume 365 ± 40 mm^3^). The treatment with DEDA at reperfusion and repeated at 3 h of reperfusion also improved neurologic functions (Fig 1C). The neurologic score of individual animal from the Veh group was 4,3,3,3,2 (severe deficit) and the animals in the treatment group (DEDA) had individual neurologic score 1,1,1,2,2 (mild deficit). We further investigated the effectiveness of a single but equivalent dose of DEDA administration at 0 h of reperfusion on infarct volume and neurologic deficit. The single dose of DEDA was found to be less effective at reducing infarct volume than the repeated dose. However, reductions in both the infarct volume (infarct volume 186 ± 25 mm^3^) and neurologic deficit (median 2.0) in the single dose of DEDA treated group were significantly improved as compared to the Veh group. The selected dose of DEDA had no significant effects on physiologic parameters (cranial temperature, mean blood pressure, heart rate and pH) as shown in Table 1.

### DEDA reduces the levels of IR-mediated release of FFA and LPC

**Figure 2:**
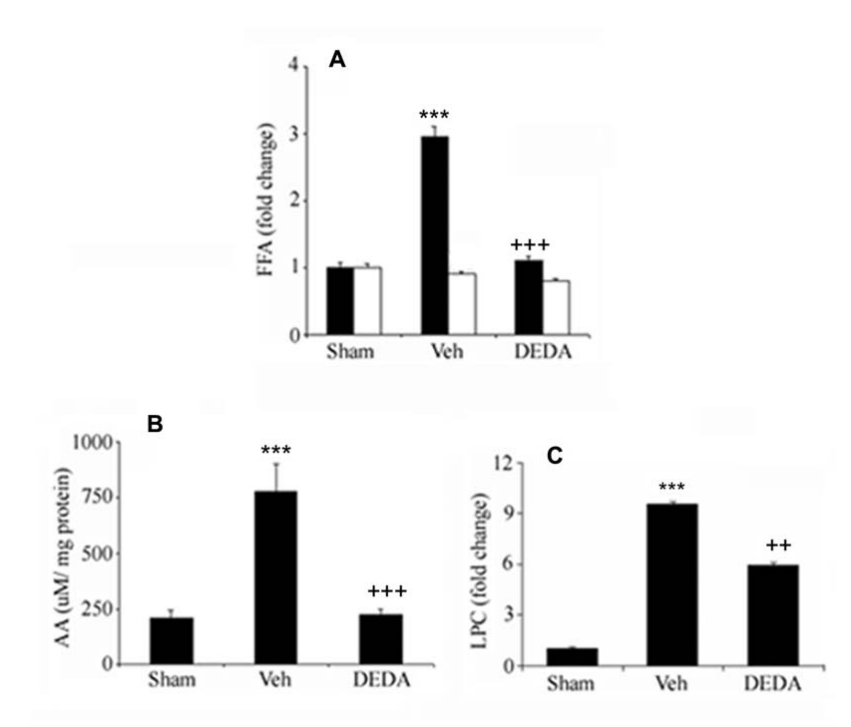
**Effect of IR injury and DEDA treatment on the levels of FFA, AA and LPC in brain tissue at 24 hours of reperfusion after 60 min MCAO**. Levels of FFA (A) and AA (B) were measured using GC and content of LPC (C) was quantitated by HPTLC. Data are expressed as means ± SD from triplicate determinations of 5 different samples (n = 5). Closed bars represent ipsilateral and open bars represent contralateral hemispheres. *** p < 0.001 vs. Sham, +++ p < 0.001 vs. Veh and ++ p < 0.01 vs. Veh.

Increases in the levels of both neurovascular toxic FFA and LPC have been documented following IR injury [29,33]. To test whether sPLA2 was responsible for altered lipid metabolism, we treated the animals with DEDA at 0 and 3 hr of reperfusion following MCAO and measured the levels of total FFA, including free AA and LPC after 24 hr of reperfusion. The treatment significantly (p < 0.001) reduced IR-mediated increased levels of total FFA (measured by HPTLC), free AA (measured by GC) and LPC (measured by HPTLC) in the penumbra region of the ipsilateral hemisphere (Fig 2A–C).

### DEDA inhibits activity of Ca^2+^-dependent PLA2s and reduces sPLA2 enzyme expression but not sPLA2 mRNA expression

**Figure 3:**
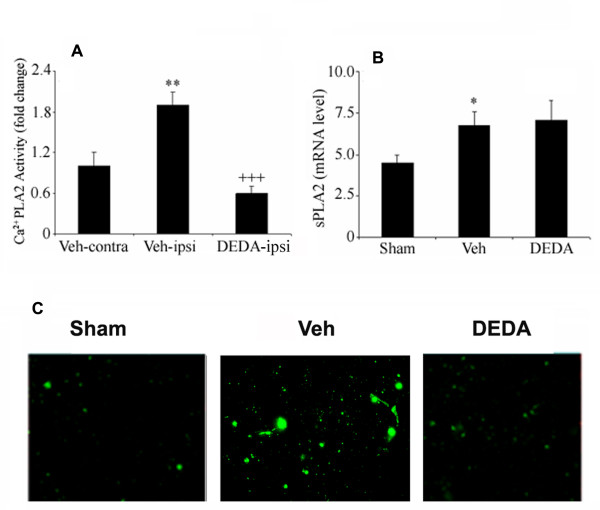
**Effect of IR injury and DEDA on Ca^2+ ^dependent PLA2 activity, mRNA and protein expression of sPLA2 in brain tissue at 24 hours of reperfusion after 60 min MCAO**. IR induced activity of calcium-dependent PLA2 was measured in both ipsilateral as well as contralateral hemispheres. Treatment with DEDA reduced the PLA2 activity (A). However, treatment with DEDA did not alter IR-induced mRNA expression of sPLA2 measured at 4 hours of reperfusion (B). Representative photomicrograph of protein expression of sPLA2 at 24 hour reperfusion (C). Data represented as means ± SD of triplicate determinations (n = 5). * p < 0.05 vs. Sham, ** p < 0.01 vs. Veh-contra, +++ p < 0.001 vs. Veh-Ipsi.

DEDA is reported as a specific and competitive inhibitor of sPLA2 group II [35]. However, its effect on the expression of sPLA2 and the activity of other calcium-dependent PLA2s is not clear. We investigated the effect of DEDA on the expression of sPLA2. Fig 3A shows that DEDA inhibited the activity of Ca^2+^-dependent PLA2s, which was significantly increased (p < 0.01) in the ipsilateral side of untreated animals compared to the contralateral. An RT-PCR study showed that the treatment with DEDA did not reduce mRNA expression measured at 4 hr after reperfusion following MCAO (Fig 3B). Enhanced expression of the sPLA2 enzyme at the protein level was observed in the penumbra region measured at 24 hr after reperfusion following MCAO using immunohistochemistry. However, it was reduced in the DEDA-treated penumbral area (Fig 3C). DEDA is an activity inhibitor of sPLA2. However, the mechanisms of DEDA's inhibition of enzyme expression are not understood.

### DEDA reduces levels of protein carbonyl and restores glutathione content

**Figure 4:**
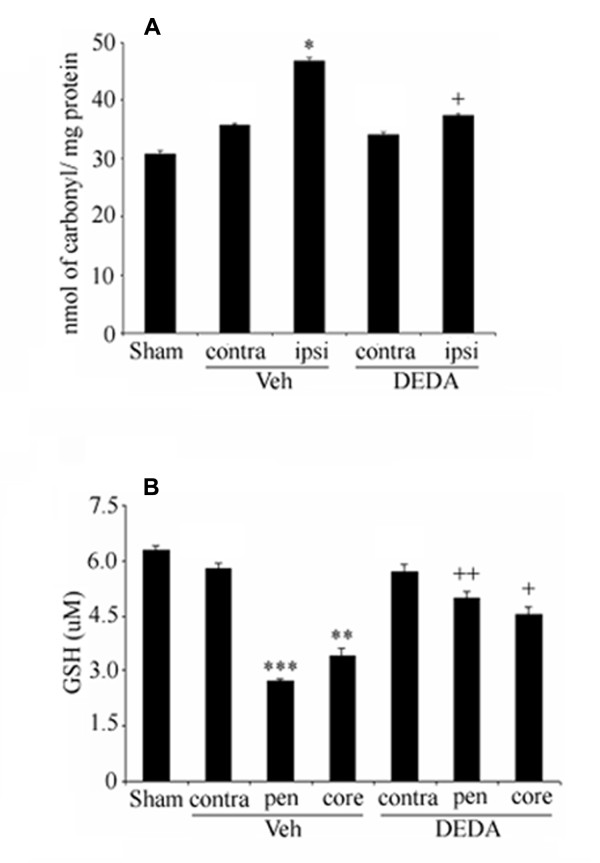
**Effect of IR and DEDA on levels of PC and GSH in brain tissue at 24 hours of reperfusion after 60 min MCAO**. Levels of protein carbonyl were significantly elevated in the ipsilateral from the Veh brain. Treatment with DEDA reduced the PC level (A). IR significantly depleted the level of GSH in the penumbra and treatment with DEDA attenuated the GSH content in the Veh brain (B). Data are expressed as means ± SD from triplicate determinations of four different samples in each group. *p < 0.05 vs. Sham, *** p < 0.001 and ** p < 0.01 vs. Sham, ++ p < 0.01 and + p < 0.05 vs. Veh.

Increased protein carbonyl formation and decreased levels of glutathione have been observed following IR injury in animal models, mainly due to ROS formation [6,34]. However, the relationship between sPLA2 activity and the imbalance of redox is not clear. To assess the effect of DEDA on oxidative stress, we measured the levels of protein carbonyls and glutathione. The level of protein carbonyls was significantly increased (p < 0.05) in the ipsilateral region from Veh animals compared to the sham. However, a non-significant increase in the contralateral side was also observed. The treatment with DEDA at 0 h and at 3 h of reperfusion significantly reduced protein carbonyl formation (Fig 4A) indicating that DEDA reduced oxidative burden following IR injury. Treatment with DEDA under similar conditions increased the levels of glutathione in the ipsilateral hemisphere (Fig 4B).

### DEDA protects BBB integrity by reducing its leakage and decreasing edema

**Figure 5:**
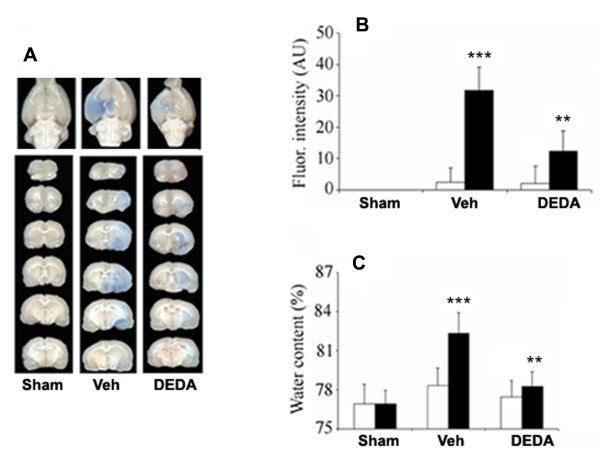
**Effect of DEDA on IR injury-induced BBB leakage and edema**. Representative photographs showing extravasation of Evan's blue (EB) in six coronal sections of brain (A). Spectrofluorimetric estimation of EB in six coronal sections (B). Significant EB leakage was observed in Veh brain and the leakage was reduced in the DEDA-treated group. EB extravasation was not observed in Sham animals. Treatment with DEDA decreased the brain water content in ipsilateral brain (C). Closed bars represent ipsilateral and open bars represent contralateral hemispheres. Data are expressed as means ± SD from triplicate determinations of three different samples in each group. *** p < 0.01 vs. Sham, ** p < 0.01 vs. Veh.

BBB disruption and edema are the hallmark of IR injury leading to inflammation and secondary injury. An assessment by the EB extravasation method showed reduced BBB leakage after DEDA treatment (Fig 5A). Measurement of fluorescence in the homogenized ipsilateral side indicated a significant decrease in EB intensity in the DEDA-treated groups compared to sham, indicating the efficacy of DEDA for BBB protection. The results were further supported by decreased edema/water content in the ipsilateral side of the DEDA treated animals as compared to the vehicle treated group (Fig 5C).

### DEDA inhibits the inflammation-mediated release of AA but does not protect against AA-induced morphological and oxidative alterations in neuronal cell lines

**Figure 6:**
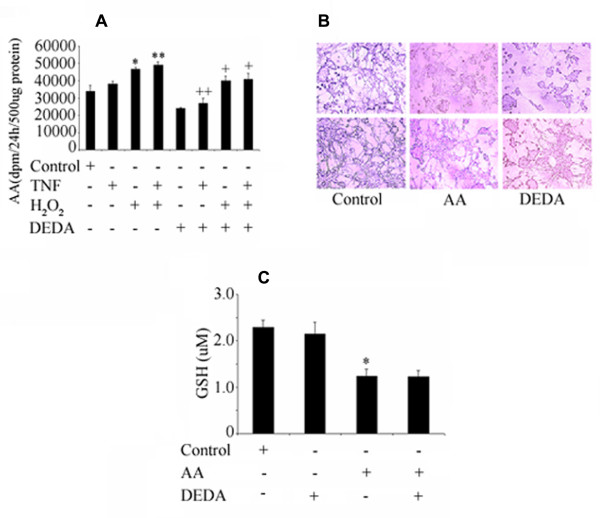
**Effect of DEDA on release of AA, cell morphology and levels of GSH in PC-12 cells**. (A) Neuronal PC12 cells were labeled with [^14^C] AA over night as described in Methods. Cells were treated with 20 μM of DEDA for 2 hour followed by treatment with TNF-α (100 ng/ml) or/and H_2_O_2 _(20 μM). After 3 h, radioactivity released in the medium (100 μl) was measured. (B) Morphology of PC12 cells after treatment with AA (50 μM) and DEDA (20 μM). (C) Effect of AA (100 μM) and DEDA (20 μM) on the GSH level. Data are expressed as means ± SD of (n = 3). * p < 0.05 and ** p < 0.01 vs. control and ++ p < 0.01 and +p < 0.05 vs. TNF-α+ H_2_O_2 _or H_2_O_2_.

IR injury induces the expression of pro-inflammatory cytokines, including TNF-α and IL-1β, leading to induction of sPLA2, generation of FFA/AA and accumulation of ROS. FFA and especially unesterified AA are significant mediators of oxidative stress, causing BBB disruption and neuronal cell death [47]. Fig 6A shows the effects of H_2_O_2 _and TNF-α on the release of AA from PC12 cells. Treatment with cytokine alone did not have a significant effect on the release of free AA. However, H_2_O_2 _alone and in combination with TNF-α significantly increased the release of AA. A pre-treatment with DEDA inhibited the release of AA remarkably. Fig 6B–C shows the effect of AA-mediated alterations in cell morphology and oxidative stress. A pre-treatment with DEDA failed to restore cell morphology. Similarly, DEDA did not restore the AA-mediated loss of GSH in these cells.

## Discussion

In experimental stroke, BBB integrity is compromised early and provides a therapeutic target of intervention [53]. Studies have shown that modulation of PLA2 and/or PLA2-derived products, including AA and LPC, has attenuated IR injury [11,47,54]. In this study, we tested the hypothesis that DEDA, an sPLA2 inhibitor, attenuates IR injury through modulation of BBB functions. The two important forms of PLA2s, cPLA2 and sPLA2, are calcium dependent and located in the cytosol. They cleave membrane phospholipids at the *sn*-2 position to yield FFA/AA and LPC. Earlier reports documented that IR injury activates these enzymes from a very early stage, continuing for several days, mainly due to increased calcium overload, which causes oxidative stress, inflammation and apoptosis [34,55,56]. Recent studies have shown the importance of sPLA2 in IR injury since it has both a tissue localized and a circulatory existence. Activation of sPLA2 leads to enhanced release of free AA and accumulation of LPC, neither of which occurs under normal circumstances. Released AA produces bioactive lipid mediators through its lipoxidative metabolisms. AA-derived oxidative lipid metabolites and LPC cause BBB leakage, endothelial dysfunction and brain edema [10,57,58].

Under normal conditions, the PLA2 enzymes help to maintain membrane composition and thus membrane integrity. However, PLA2s produce excessive amount of FFA/AA and LPC under pathological condition. A high amount of accumulated LPC, as observed in IR injury (Fig 2C), may cause electrophysiological disturbances [59] and hence membrane dysfunction. Post-ischemia treatment with AA aggravates IR-induced BBB leakage and water retention, enhancing oxidative stress through depletion of GSH and increasing MDA levels [56]. IR is accompanied by generation of ROS/reactive nitrogen species (RNS) and inflammation. Several reports show the beneficial effects of anti-inflammatory therapy after IR injury. Most are based on the inhibition of LOX and COX-2 enzymes, thus preventing the conversion of AA to prostaglandins. However, studies on AA confirm that it has direct deleterious effects itself during IR injury through other mechanisms as well [14]. Therefore, inhibiting enhanced AA/LPC release may be a more effective therapeutic strategy over those that retard AA conversion to other inflammatory mediators. Elevated concentrations of FFA also contribute to enhanced expression of monocytes and their adhesion to endothelial cells [60], which may lead to BBB leakage. In the present study, we observed the inhibitory effect of DEDA on sPLA2 activity, which is responsible for excessive FFA/AA and LPC release during IR injury.

In ischemic conditions, the initial injury in the core area is caused mainly by necrotic neuronal cell death due to the lack of oxygen and the depletion of energy. The cellular death during reperfusion is primarily apoptotic in nature, which is also an energy dependent process. This apoptosis expands the core injury, converting the penumbra previously amenable to therapeutic intervention into a larger and permanently damaged infarct [41]. AA has been reported to promote apoptotic cell death by enhancing caspase-3 and myeloperoxidase (MPO) activity [56]. On the other hand, accumulated LPC acts like a detergent and helps to make the membrane more soluble [59]. The BBB is a neurovascular system mainly composed of endothelial and astroglial cells with a basal lamina. It is highly discriminatory, and has selective permeability inside the brain. These traits of the BBB suggest that the combined effect of AA and LPC may be disruptive, and disruption of BBB is one of the major causes of secondary injury. In this disruption, unwanted cells, debris and water transmigrate across and infiltrate the BBB, which finally leads to vasogenic edema, a prominent cause of mortality following IR injury.

Our findings show that DEDA significantly reduced the infarct size and improved neurologic score (Fig 1A–C). This effect may occur through inhibition of FFA/AA and LPC release (Fig 2A–C). Fig 3A clearly indicates that, even though DEDA down regulated PLA2 activity, it did not have any significant effect on the expression of PLA2 at mRNA levels (Fig 3B–C).

The mechanism of IR injury is complex and multi-factorial. Among these factors is oxidative stress, which regulates neuroinflammation [34,44]. An imbalance in the oxidant-antioxidant homeostasis can be observed by studying GSH level. Fig 4A–B shows that IR injury resulted in oxidative stress; excessive AA may be one of the major culprits, as PLA2 is activated within minutes following stroke in different cell type including reactive astrocytes [31]. On one hand, AA weakens the endogenous antioxidant system represented by GSH levels; on the other hand, it enhances formation of lipid metabolites, which further interact with various proteins, thus forming protein carbonyl. Carbonylation of proteins makes them inaccessible to specific enzymes by altering their identity and hence hindering their function and degradation. Supporting the efficacy of DEDA, Fig 4A–B shows that treatment with DEDA significantly attenuated the oxidative stress due to IR injury. To further confirm the relationship between excessive AA and oxidative stress, we studied an in vitro model by treating the neuronal cells with H_2_O_2 _and AA. Fig 6A shows that there is significantly increased AA release, possibly resulting from activation of PLA2 due to oxidative stress. DEDA significantly attenuated the altered release of AA. Furthermore, Fig 6B–D shows that AA and other lipid metabolites like 4-hydroxynonenal (4-HNE) induced morphological changes and depleted GSH. GSH plays significant role in stroke and its restoration by its precursor NAC [7] or by CDP-choline [61] is associated with neurovascular protection. These findings show that the induction of oxidative stress occurs through lipid metabolites, and this phenomenon helps to activate PLA2, contributing to secondary injury. It also confirms that DEDA may provide protection in IR injury by reducing oxidative stress through modulation of PLA2 activity and excessive AA release. However, DEDA clearly does not have direct antioxidant properties to combat oxidative stress.

Earlier reports documented that AA leads to BBB dysfunction and edema [62,63]; it is possible that exogenously accumulated LPC with a membrane-lytic activity may play a synergistic role with AA. Fig 5A–B shows EB leakage inside the brain as a marker of BBB disruption and dysfunction. This compromised BBB integrity leads to edema due to an influx of water and other contents (Fig 5C). Inhibition of PLA2 activity by DEDA in the acute phase significantly reduced BBB leakage and edema formation. BBB leakage and edema formation may have life-threatening consequences, worsening neurologic outcomes. Preservation of BBB integrity by DEDA treatment indicates that the use of a PLA2 inhibitor in the acute phase of IR injury may have favorable therapeutic outcomes.

## Conclusion

The present study found that release of FFA/AA and LPC are among the few critical earlier events of IR injury. Excessive release of AA and LPC by sPLA2 leads to BBB dysfunction, inflammation and oxidative stress, which in turn, cause secondary injury. Moreover, induction of sPLA2 and the consequent accumulation of LPC and AA-metabolites may compromise membrane integrity following IR injury. Therefore, an inhibition of sPLA2 by DEDA or other pharmacological means may protect BBB integrity and provide significant therapeutic benefits following stroke.

## List of Abbreviations

AA: Arachidonic acid; BBB: Blood brain barrier; COX: Cycloxygenase; DEDA: 7,7'-Dimethyl eicosadienoic acid; EB: Evan's blue; FFA: Free fatty acids; GC: Gas chromatography; GPx: Glutathione peroxidase; GST: Glutathione S-transferase; GSH: Reduced glutathione; 4-HNE: 4-Hydroxynonenal; HPTLC: High performance thin layer chromatography; H_2_O_2_: Hydrogen peroxide; IR: Ischemia-reperfusion; LOX: Lipoxygenase; LPC: Lysophosphatidylcholine; MCAO: Middle cerebral artery occlusion; MDA: Malondialdehyde; MPO: Myeloperoxidase; NAC: N-acetylcysteine; PC: Protein carbonyl; PCR: Polymerase chain reaction; RNS: Reactive nitrogen species; ROS: Reactive oxygen species; sPLA2: Secretory phospholipases A2; TCA: Trichloro acetic acid; TNF: Tumor necrosis factor; Veh: Vehicle.

## Competing interests

The authors declare that they have no competing interests.

## Authors' contributions

This study is based on an original idea of MK and IS. MK and NH wrote the manuscript. NH and MK carried out animal and biochemical studies. AKS critically examined histochemical studies and corrected the manuscript. All authors have read and approved the manuscript.

## References

[B1] Chan PH (2001). Reactive oxygen radicals in signaling and damage in the ischemic brain. J Cereb Blood Flow Metab.

[B2] Mehta SL, Manhas N, Raghubir R (2007). Molecular targets in cerebral ischemia for developing novel therapeutics. Brain Res Rev.

[B3] Lindsay T, Walker PM, Mickle DA, Romaschin AD (1988). Measurement of hydroxy-conjugated dienes after ischemia-reperfusion in canine skeletal muscle. Am J Physiol.

[B4] O'Regan MH, Song D, Heide SJ Vander, Phillis JW (1997). Free radicals and the ischemia-evoked extracellular accumulation of amino acids in rat cerebral cortex. Neurochem Res.

[B5] Lewen A, Matz P, Chan PH (2000). Free radical pathways in CNS injury. J Neurotrauma.

[B6] Warner DS, Sheng H, Batinic-Haberle I (2004). Oxidants, antioxidants and the ischemic brain. J Exp Biol.

[B7] Khan M, Sekhon B, Jatana M, Giri S, Gilg AG, Sekhon C, Singh I, Singh AK (2004). Administration of N-acetylcysteine after focal cerebral ischemia protects brain and reduces inflammation in a rat model of experimental stroke. J Neurosci Res.

[B8] Lambeau G, Gelb MH (2008). Biochemistry and physiology of mammalian secreted phospholipases A2. Annu Rev Biochem.

[B9] Phillis JW, O'Regan MH (2003). The role of phospholipases, cyclooxygenases, and lipoxygenases in cerebral ischemic/traumatic injuries. Crit Rev Neurobiol.

[B10] Adibhatla RM, Hatcher JF (2007). Secretory phospholipase A2 IIA is up-regulated by TNF-alpha and IL-1alpha/beta after transient focal cerebral ischemia in rat. Brain Res.

[B11] Yagami T, Ueda K, Asakura K, Hata S, Kuroda T, Sakaeda T, Takasu N, Tanaka K, Gemba T, Hori Y (2002). Human group IIA secretory phospholipase A2 induces neuronal cell death via apoptosis. Mol Pharmacol.

[B12] Sun GY, Xu J, Jensen MD, Simonyi A (2004). Phospholipase A2 in the central nervous system: implications for neurodegenerative diseases. J Lipid Res.

[B13] Marnett LJ (1994). Generation of mutagens during arachidonic acid metabolism. Cancer Metastasis Rev.

[B14] Scorrano L, Penzo D, Petronilli V, Pagano F, Bernardi P (2001). Arachidonic acid causes cell death through the mitochondrial permeability transition. Implications for tumor necrosis factor-alpha aopototic signaling. J Biol Chem.

[B15] Toborek M, Malecki A, Garrido R, Mattson MP, Hennig B, Young B (1999). Arachidonic acid-induced oxidative injury to cultured spinal cord neurons. J Neurochem.

[B16] Vento R, D'Alessandro N, Giuliano M, Lauricella M, Carabillo M, Tesoriere G (2000). Induction of apoptosis by arachidonic acid in human retinoblastoma Y79 cells: involvement of oxidative stress. Exp Eye Res.

[B17] Becuwe P, Bianchi A, Didelot C, Barberi-Heyob M, Dauca M (2003). Arachidonic acid activates a functional AP-1 and an inactive NF-kappaB complex in human HepG2 hepatoma cells. Free Radic Biol Med.

[B18] Camandola S, Leonarduzzi G, Musso T, Varesio L, Carini R, Scavazza A, Chiarpotto E, Baeuerle PA, Poli G (1996). Nuclear factor kB is activated by arachidonic acid but not by eicosapentaenoic acid. Biochem Biophys Res Commun.

[B19] Maziere C, Conte MA, Degonville J, Ali D, Maziere JC (1999). Cellular enrichment with polyunsaturated fatty acids induces an oxidative stress and activates the transcription factors AP1 and NFkappaB. Biochem Biophys Res Commun.

[B20] Nito C, Kamada H, Endo H, Niizuma K, Myer DJ, Chan PH (2008). Role of the p38 mitogen-activated protein kinase/cytosolic phospholipase A2 signaling pathway in blood-brain barrier disruption after focal cerebral ischemia and reperfusion. J Cereb Blood Flow Metab.

[B21] Shimizu T, Wolfe LS (1990). Arachidonic acid cascade and signal transduction. J Neurochem.

[B22] Farooqui AA, Yang HC, Rosenberger TA, Horrocks LA (1997). Phospholipase A2 and its role in brain tissue. J Neurochem.

[B23] Verity MA (1993). Mechanisms of phospholipase A2 activation and neuronal injury. Ann N Y Acad Sci.

[B24] Gorovetz M, Schwob O, Krimsky M, Yedgar S, Reich R (2008). MMP production in human fibrosarcoma cells and their invasiveness are regulated by group IB secretory phospholipase A2 receptor-mediated activation of cytosolic phospholipase A2. Front Biosci.

[B25] Hung YC, Chen TY, Lee EJ, Chen WL, Huang SY, Lee WT, Lee MY, Chen HY, Wu TS (2008). Melatonin decreases matrix metalloproteinase-9 activation and expression and attenuates reperfusion-induced hemorrhage following transient focal cerebral ischemia in rats. J Pineal Res.

[B26] Farooqui AA, Horrocks LA (2004). Brain phospholipases A2: a perspective on the history. Prostaglandins Leukot Essent Fatty Acids.

[B27] Fuentes L, Hernandez M, Nieto ML, Sanchez Crespo M (2002). Biological effects of group IIA secreted phosholipase A(2). FEBS Lett.

[B28] Kudo I, Murakami M (2002). Phospholipase A2 enzymes. Prostaglandins Other Lipid Mediat.

[B29] Farooqui AA, Ong WY, Horrocks LA (2006). Inhibitors of brain phospholipase A2 activity: their neuropharmacological effects and therapeutic importance for the treatment of neurologic disorders. Pharmacol Rev.

[B30] Reid RC (2005). Inhibitors of secretory phospholipase A2 group IIA. Curr Med Chem.

[B31] Lin TN, Wang Q, Simonyi A, Chen JJ, Cheung WM, He YY, Xu J, Sun AY, Hsu CY, Sun GY (2004). Induction of secretory phospholipase A2 in reactive astrocytes in response to transient focal cerebral ischemia in the rat brain. J Neurochem.

[B32] Hope WC, Chen T, Morgan DW (1993). Secretory phospholipase A2 inhibitors and calmodulin antagonists as inhibitors of cytosolic phospholipase A2. Agents Actions.

[B33] Pilitsis JG, Diaz FG, O'Regan MH, Phillis JW (2002). Differential effects of phospholipase inhibitors on free fatty acid efflux in rat cerebral cortex during ischemia-reperfusion injury. Brain Res.

[B34] Muralikrishna Adibhatla R, Hatcher JF (2006). Phospholipase A2, reactive oxygen species, and lipid peroxidation in cerebral ischemia. Free Radic Biol Med.

[B35] Cohen N, Weber G, Banner BL, Welton AF, Hope WC, Crowley H, Anderson WA, Simko BA, O'Donnell M, Coffey JW (1984). Analogs of arachidonic acid methylated at C-7 and C-10 as inhibitors of leukotriene biosynthesis. Prostaglandins.

[B36] Sargent CA, Vesterqvist O, McCullough JR, Ogletree ML, Grover GJ (1992). Effect of the phospholipase A2 inhibitors quinacrine and 7,7-dimethyleicosadienoic acid in isolated globally ischemic rat hearts. J Pharmacol Exp Ther.

[B37] Phillis JW, O'Regan MH (1996). Mechanisms of glutamate and aspartate release in the ischemic rat cerebral cortex. Brain Res.

[B38] Giri S, Khan M, Rattan R, Singh I, Singh AK (2006). Krabbe disease: psychosine-mediated activation of phospholipase A2 in oligodendrocyte cell death. J Lipid Res.

[B39] Longa EZ, Weinstein PR, Carlson S, Cummins R (1989). Reversible middle cerebral artery occlusion without craniectomy in rats. Stroke.

[B40] Belayev L, Alonso OF, Busto R, Zhao W, Ginsberg MD (1996). Middle cerebral artery occlusion in the rat by intraluminal suture. Neurological and pathological evaluation of an improved model. Stroke.

[B41] Khan M, Elango C, Ansari MA, Singh I, Singh AK (2007). Caffeic acid phenethyl ester reduces neurovascular inflammation and protects rat brain following transient focal cerebral ischemia. J Neurochem.

[B42] Huang Z, Huang PL, Panahian N, Dalkara T, Fishman MC, Moskowitz MA (1994). Effects of cerebral ischemia in mice deficient in neuronal nitric oxide synthase. Science.

[B43] Jatana M, Giri S, Ansari MA, Elango C, Singh AK, Singh I, Khan M (2006). Inhibition of NF-kappaB activation by 5-lipoxygenase inhibitors protects brain against injury in a rat model of focal cerebral ischemia. J Neuroinflammation.

[B44] Khan M, Sekhon B, Giri S, Jatana M, Gilg AG, Ayasolla K, Elango C, Singh AK, Singh I (2005). S-Nitrosoglutathione reduces inflammation and protects brain against focal cerebral ischemia in a rat model of experimental stroke. J Cereb Blood Flow Metab.

[B45] Khan M, Singh J, Singh I (2008). Plasmalogen deficiency in cerebral adrenoleukodystrophy and its modulation by lovastatin. J Neurochem.

[B46] Khan M, Contreras M, Singh I (2000). Endotoxin-induced alterations of lipid and fatty acid compositions in rat liver peroxisomes. J Endotoxin Res.

[B47] Rao AM, Hatcher JF, Kindy MS, Dempsey RJ (1999). Arachidonic acid and leukotriene C4: role in transient cerebral ischemia of gerbils. Neurochem Res.

[B48] Weerheim AM, Kolb AM, Sturk A, Nieuwland R (2002). Phospholipid composition of cell-derived microparticles determined by one-dimensional high-performance thin-layer chromatography. Anal Biochem.

[B49] Adibhatla RM, Hatcher JF (2003). Citicoline decreases phospholipase A2 stimulation and hydroxyl radical generation in transient cerebral ischemia. J Neurosci Res.

[B50] Paintlia AS, Paintlia MK, Singh AK, Stanislaus R, Gilg AG, Barbosa E, Singh I (2004). Regulation of gene expression associated with acute experimental autoimmune encephalomyelitis by Lovastatin. J Neurosci Res.

[B51] Levine RL, Garland D, Oliver CN, Amici A, Climent I, Lenz AG, Ahn BW, Shaltiel S, Stadtman ER (1990). Determination of carbonyl content in oxidatively modified proteins. Methods Enzymol.

[B52] Weissman DE, Stewart C (1988). Experimental drug therapy of peritumoral brain edema. J Neurooncol.

[B53] Strbian D, Durukan A, Pitkonen M, Marinkovic I, Tatlisumak E, Pedrono E, Abo-Ramadan U, Tatlisumak T (2008). The blood-brain barrier is continuously open for several weeks following transient focal cerebral ischemia. Neuroscience.

[B54] Watanabe T, Egawa M (1994). Effects of an antistroke agent MCl-186 on cerebral arachidonate cascade. J Pharmacol Exp Ther.

[B55] Adibhatla RM, Hatcher JF, Larsen EC, Chen X, Sun D, Tsao FH (2006). CDP-choline significantly restores phosphatidylcholine levels by differentially affecting phospholipase A2 and CTP: phosphocholine cytidylyltransferase after stroke. J Biol Chem.

[B56] Yang DY, Pan HC, Yen YJ, Wang CC, Chuang YH, Chen SY, Lin SY, Liao SL, Raung SL, Wu CW (2007). Detrimental effects of post-treatment with fatty acids on brain injury in ischemic rats. Neurotoxicology.

[B57] Chan PH, Fishman RA (1978). Brain edema: induction in cortical slices by polyunsaturated fatty acids. Science.

[B58] Smith WL, Garavito RM, DeWitt DL (1996). Prostaglandin endoperoxide H synthases (cyclooxygenases)-1 and -2. J Biol Chem.

[B59] O'Regan MH, Perkins LM, Phillis JW (1995). Arachidonic acid and lysophosphatidylcholine modulate excitatory transmitter amino acid release from the rat cerebral cortex. Neurosci Lett.

[B60] Zhang WY, Schwartz E, Wang Y, Attrep J, Li Z, Reaven P (2006). Elevated concentrations of nonesterified fatty acids increase monocyte expression of CD11b and adhesion to endothelial cells. Arterioscler Thromb Vasc Biol.

[B61] Adibhatla RM, Hatcher JF, Dempsey RJ (2001). Effects of citicoline on phospholipid and glutathione levels in transient cerebral ischemia. Stroke.

[B62] Papadopoulos SM, Black KL, Hoff JT (1989). Cerebral edema induced by arachidonic acid: role of leukocytes and 5-lipoxygenase products. Neurosurgery.

[B63] Unterberg A, Wahl M, Hammersen F, Baethmann A (1987). Permeability and vasomotor response of cerebral vessels during exposure to arachidonic acid. Acta Neuropathol.

